
JOURNAL INFORMATION
==============================
NLM Title Abbreviation: J Can Health Libr Assoc
Journal ID: J Can Health Libr Assoc
NLM Journal ID: 101229936
Journal ID: JCHLA

The Journal of the Canadian Health Libraries Association

EISSN: 1708-6892
Publisher: Canadian Health Libraries Association / Association des bibliotèques de la santé du Canada

ARTICLE INFORMATION
==============================
PMCID: PMC11485153
DOI: 10.29173/jchla29788
Article ID: JCHLA-45-127
Article version: 1
Subjects: Chla 2024 Conference Posters / Absc Congrès 2024 Affiches

CHLA 2024 CONFERENCE POSTERS / ABSC CONGRÈS 2024 AFFICHES

Electronic publication date: 2024 Aug 1
Publication date: 2024 Aug
Volume: 45
Issue: 2
First page: 127
Last page: 130
License: This article is distributed under a Creative Commons Attribution License: https://creativecommons.org/licenses/by/4.0/
License URL: https://creativecommons.org/licenses/by/4.0/

==============================

PP = Poster Presentation

## PP1. Advocating for equitable access: using survey data to identify system wide strategies for equitable access to library resources

Benjamin Hoover, Kelly Thormodson
Penn State University

**Objective:** The Penn State College of Medicine and Penn State Health have separate medical libraries and changes have made it difficult to determine who has access to either library and their very different collections. What strategies have other health sciences libraries implemented to provide/extend equitable access to the library information resources and services to affiliated health system members and which of the identified strategies would be most applicable to the research teams' institution? **Methods:** This project utilizes survey data collected in 2022 from health sciences libraries across North America. That survey and subsequent publication were focused on the challenges and opportunities for libraries in relation to fast growing academic health systems. Additionally, an updated literature search will be conducted. **Results:** From this survey data set, the research team has identified relevant questions focused on what educational groups and hospital/health systems do the libraries serve and what strategies are used to integrate hospital/health systems to provide services and access to library information resources. The team will focus on these questions and analyze responses to identify patterns in how health sciences libraries provide services to health systems and related academic institutions. **Discussion:** The analysis will include reviewing both common and novel approaches that could be applicable to the research teams' intuitional structure. The final step of this research project is to identify and select up to two service/access models for the library administration to advocate as the best for the users, the library, and the systems.

## PP2. Are patrons "clicking" with the library's literature search service? Assessing patron engagement using Short.io

Tyler Ostapyk, Carla Epp, Nicole Askin
University of Manitoba

**Introduction:** One of the core services the Winnipeg Regional Health Authority Virtual Library provides is literature searching for healthcare staff. Results are sent to patrons as a reference list in Word format. These lists contain links to items available freely to the public or through the library's collection. Library staff wanted to track if and how patrons were engaging with items in the lists they received. **Methods:** Leveraging the link management tool Short.io and a Visual Basic for Applications macro, an automated process was developed to generate and add trackable links to the reference lists sent to patrons. Click data was automatically collected in Short.io and data for links created from October 17, 2022-October 17, 2023 was exported into Excel for analysis. **Results:** As of October 17, 2023, 169 literature searches were sent to patrons using this method. Patrons clicked at least one link in 54% of reference lists. UpToDate links had a click rate of 42% and JBI EBP links had a click rate of 45%. In contrast, 9% of DOI links, 2% of PMID links, and 8% of PMCID links were clicked. Click counts were highest for lists containing 20-30 references. **Discussion:** This data provides valuable insights into the library's literature search service. The results suggest that literature searches best meet the needs of the library's patrons when they contain

20-30 references and include links to quick reference resources. The discovery that 46% of reference lists did not have a single click warrants further investigation.

## PP3. Demonstrating library value: developing a practical tool

Nicole Askin[1], Joanne R. Hodder[2], Mark Mueller[3], Jeanna Hough[4], Brooke Scott[5], Joan Bartlett[6]
[1]University of Manitoba; [2]Nova Scotia Health; [3]Saskatchewan Health Authority; [4]Halton Health; [5]Fraser Health; [6]McGill University

**Background:** The Levels of Library Service was a proof-of-concept service visualization originally developed by the Measuring Value task force of the Health Science Information Consortium of Toronto as part of their larger Library Value Toolkit. The CHLA/ABSC Standards Standing Committee (SSC) was mandated to develop practical tools to help libraries apply the CHLA/ABSC Standards, building on the Library Value Toolkit. **Description:** The SSC reimagined the Levels of Library Service into a dynamic checklist that can be customized to map library services and the associated resource requirements for the purposes of operational and strategic planning. A preliminary draft of this new tool was shared with community members via focus groups and a national survey as part of a broader SSC website. **Outcomes:** The SSC is currently in the process of revising the website and tool based on the feedback received. The SSC will create educational materials to support libraries in adopting the tool in their contexts.

## PP4. Exploring donor relationship management in academic and health sciences libraries: a pilot survey on scale purification

Like other nonprofits, health sciences libraries rely increasingly on philanthropic support to fulfill their missions. However, unlike public libraries, limited research exists on how these libraries engage with donors effectively. Using an established conceptual framework for relationship measurement (Hon & Grunig, 1999; Jo et al., 2004; DeVellis, 2012), we seek to identify how the four pivotal dimensions crucial to donor relationship management - trust, control mutuality, commitment, and satisfaction - are applied within the unique context of libraries, in particular health sciences libraries.

Using the validated scales developed by Hon & Grunig, we will employ a meticulous judgmental approach, to assess items for relevance, logic, and inclusiveness, within the field of health sciences libraries. Seeking input from library conference attendees in both Canada and the United States, we intend to pilot-test the survey, using a method called scale purification. Guided by Moore and Benbasat's (1991) three-stage framework, the research progresses through the iterative processes of item pool creation, instrument development, and rigorous instrument testing. The goal is to develop a comprehensive and reliable tool for assessing donor relationships in academic and health sciences libraries. The study sets the stage for a forthcoming full-scale survey, offering insights that can inform library professionals, administrators, and others on optimizing donor engagement strategies for sustainable support.

This is the first step in a multi-year project to assess donor relationships in health libraries. Further research will include a full-scale survey of library professionals and focus groups of persons engaged with donor relations in academic and health sciences libraries.

## PP5. Filtering failure: use of exp animals/ not humans.sh with automated indexing

Nicole Askin, Tyler Ostapyk, Carla Epp
University of Manitoba

**Introduction:** The search string ‘exp animals/ not humans.sh’ is a well-established method in evidence synthesis used to exclude non-human studies in Ovid Medline. However, the shift to automatic indexing of Medline records has raised concerns about the use of subject-heading-based search techniques. **Methods:** We conducted a search in Ovid Medline using the Cochrane Highly Sensitive Search Strategy and identified the results excluded specifically by the non-human-studies string. These were divided into sets based on indexing method: automated, curated, or manual. Each set was screened to identify human studies. **Results:** The rate of human clinical randomized controlled trials (RCTs) excluded in the automatic set was six times that of the curated or manual sets; non-RCTs relating to humans were also incorrectly excluded. In some cases, this was the result of a single paper describing both human and non-human work, where an animals/ heading was correctly applied but a humans/ heading was absent. However, we also identified situations in which a humans/ heading was absent and an animals/ heading was also incorrect - for example, where a paper briefly mentioned prior animal work as background to the current study, or where an abstract simply used animal-related language (eg. “participants were instructed to click the mouse”). **Discussion:** Some records were updated during our project to add absent humans/ headings, but the error rate remains significant. Given our findings, we recommend against continued use of the exp animals/ not humans.sh search string, pending improvements to the automated indexing process.

## PP6. MeSHing around with citation metadata to explore patterns in automatic indexing: preliminary results

Emma Garlock, Joan Bartlett
McGill University

**Introduction:** The Medical Text Indexer (MTI) has been incredibly impactful, with a notable decrease in the time it takes a MEDLINE citation to receive MeSH indexing. However, further work is needed to address some well documented issues around the indexing genes and chemical compounds, and their impact on information retrieval. To investigate these issues, this research pursues the following research questions: RQ1. Is there a relationship between indexing method or journal impact factor (JIF) and how well do MeSH terms align with keywords and chemical symbols? RQ2. Is there a relationship between indexing method or JIF and the term usage frequencies among MeSH, keywords, and chemical symbols? **Methods:** The research method analyzed the indexing in a sample of MEDLINE citations. 648 citations published between January 2021 and December 2023 were randomly selected and relevant information fields extracted via NLM’s efetch and xtract tools. Journal impact factor data was downloaded from Clarivate. Using R, a n-gram analysis and the relative frequency of each term will address RQ1 and RQ2, respectively. **Results:** Preliminary results and interpretation of results for RQ1 using an n-gram word model and relative frequency results for RQ2 will be presented. **Discussion:** We will discuss the results of comparing term alignment in the context of information retrieval and term use frequency in the context of MTI performance and optimization. Overall implications for information retrieval and instruction in a health libraries context will be addressed.

## PP7. Social prescribing: a tool for primary care

Taylor Moore
Centre for Effective Practice

**Introduction:** In October 2023, the Centre for Effective Practice (CEP) launched a new web-based tool: Social Prescribing: A Resource for Health Professionals. The social prescribing model in primary care aims to address the social determinants of health that can impact an individual's well-being. **Description:** While undertaking the scoping work, the content development team discovered that there was limited evidence on the impact and processes of social prescribing in primary care practices across Canada. Instead of relying on evidence, which is typically how CEP tools are developed, the team had to pivot and develop partnerships with leaders in the social prescribing community in Ontario and across Canada, as well as with lived experience experts. **Outcomes:** As of January 2024, there have been over 2,800 downloads of the tool. The CEP team presented the tool at a social prescribing conference in October 2023, and is planning future tools in partnership with external stakeholders including the Alliance for Healthier Communities, the Canadian Institute for Social Prescribing, and others. **Discussion:** CEP's tool is the first of its kind to deliver practical social prescribing tips for primary care clinicians. The tool is applicable in a wide variety of primary care settings across Ontario and can easily be adapted to fit the specific needs of clinicians in all types of practices. While working on the tool, the CEP team benefited greatly from partnerships with key stakeholders in the social prescribing community, paving the way for even more opportunities to create other digital tools in the future.

## PP8. Supporting primary care clinicians with evidence-based digital tools

Taylor Moore
Centre for Effective Practice

**Introduction:** The Centre for Effective Practice (CEP) is one of the leading independent healthcare behaviour-change partners in Canada, and a trusted source of high-quality, evidence-based clinical supports for primary care clinicians. To assist with the CEP's mission to close the gap between evidence and practice, CEP's in-house medical librarians (the Information Services team) are responsible for the evidence searching, appraisal and curation, as well as critical analysis, synthesis and knowledge translation. **Description:** The Information Services team supports CEP projects in a variety of ways, with the scope and depth of team involvement based on the project's stream of work and needs. The team's primary responsibilities are to provide evidence throughout the development of a project, to support knowledge translation, and to provide content for CEP's web-based and EMR tools. CEP's projects are primarily divided into three streams: Knowledge Translation in Primary Care (KTinPC), Evidence2Practice (E2P), and market-based services. **Outcomes**: Overall, 98% of clinicians report that CEP's tools improve knowledge while 99% of clinicians say that CEP translates evidence into practical patient care. **Discussion:** The Information Services team at CEP is involved in projects that go beyond traditional library roles. The librarians are considered the evidence experts in the organization, and they play an integral part in the development of tools to assist primary care clinicians.

JOURNAL INFORMATION
==============================
NLM Title Abbreviation: J Can Health Libr Assoc
Journal ID: J Can Health Libr Assoc
NLM Journal ID: 101229936
Journal ID: JCHLA

The Journal of the Canadian Health Libraries Association

EISSN: 1708-6892
Publisher: Canadian Health Libraries Association / Association des bibliotèques de la santé du Canada

ARTICLE INFORMATION
==============================
Subjects: PP = Poster Presentation

PP1. Advocating for equitable access: using survey data to identify system wide strategies for equitable access to library resources

Hoover Benjamin
Thormodson Kelly
Penn State University

JOURNAL INFORMATION
==============================
NLM Title Abbreviation: J Can Health Libr Assoc
Journal ID: J Can Health Libr Assoc
NLM Journal ID: 101229936
Journal ID: JCHLA

The Journal of the Canadian Health Libraries Association

EISSN: 1708-6892
Publisher: Canadian Health Libraries Association / Association des bibliotèques de la santé du Canada

ARTICLE INFORMATION
==============================
Subjects: PP = Poster Presentation

PP2. Are patrons “clicking” with the library’s literature search service? Assessing patron engagement using Short.io

Ostapyk Tyler
Epp Carla
Askin Nicole
University of Manitoba

JOURNAL INFORMATION
==============================
NLM Title Abbreviation: J Can Health Libr Assoc
Journal ID: J Can Health Libr Assoc
NLM Journal ID: 101229936
Journal ID: JCHLA

The Journal of the Canadian Health Libraries Association

EISSN: 1708-6892
Publisher: Canadian Health Libraries Association / Association des bibliotèques de la santé du Canada

ARTICLE INFORMATION
==============================
Subjects: PP = Poster Presentation

PP3. Demonstrating library value: developing a practical tool

Askin Nicole 1
Hodder Joanne R. 2
Mueller Mark 3
Hough Jeanna 4
Scott Brooke 5
Bartlett Joan 6
1 University of Manitoba;
2 Nova Scotia Health;
3 Saskatchewan Health Authority;
4 Halton Health;
5 Fraser Health;
6 McGill University

JOURNAL INFORMATION
==============================
NLM Title Abbreviation: J Can Health Libr Assoc
Journal ID: J Can Health Libr Assoc
NLM Journal ID: 101229936
Journal ID: JCHLA

The Journal of the Canadian Health Libraries Association

EISSN: 1708-6892
Publisher: Canadian Health Libraries Association / Association des bibliotèques de la santé du Canada

ARTICLE INFORMATION
==============================
Subjects: PP = Poster Presentation

PP4. Exploring donor relationship management in academic and health sciences libraries: a pilot survey on scale purification

Ross-White Amanda 1
Iansavitchene Alla 2
1 Queen’s University
2 London Health Sciences Centre

JOURNAL INFORMATION
==============================
NLM Title Abbreviation: J Can Health Libr Assoc
Journal ID: J Can Health Libr Assoc
NLM Journal ID: 101229936
Journal ID: JCHLA

The Journal of the Canadian Health Libraries Association

EISSN: 1708-6892
Publisher: Canadian Health Libraries Association / Association des bibliotèques de la santé du Canada

ARTICLE INFORMATION
==============================
Subjects: PP = Poster Presentation

PP5. Filtering failure: use of exp animals/ not humans.sh with automated indexing

Askin Nicole
Ostapyk Tyler
Epp Carla
University of Manitoba

JOURNAL INFORMATION
==============================
NLM Title Abbreviation: J Can Health Libr Assoc
Journal ID: J Can Health Libr Assoc
NLM Journal ID: 101229936
Journal ID: JCHLA

The Journal of the Canadian Health Libraries Association

EISSN: 1708-6892
Publisher: Canadian Health Libraries Association / Association des bibliotèques de la santé du Canada

ARTICLE INFORMATION
==============================
Subjects: PP = Poster Presentation

PP6. MeSHing around with citation metadata to explore patterns in automatic indexing: preliminary results

Garlock Emma
Bartlett Joan
McGill University

JOURNAL INFORMATION
==============================
NLM Title Abbreviation: J Can Health Libr Assoc
Journal ID: J Can Health Libr Assoc
NLM Journal ID: 101229936
Journal ID: JCHLA

The Journal of the Canadian Health Libraries Association

EISSN: 1708-6892
Publisher: Canadian Health Libraries Association / Association des bibliotèques de la santé du Canada

ARTICLE INFORMATION
==============================
Subjects: PP = Poster Presentation

PP7. Social prescribing: a tool for primary care

Moore Taylor
Centre for Effective Practice

JOURNAL INFORMATION
==============================
NLM Title Abbreviation: J Can Health Libr Assoc
Journal ID: J Can Health Libr Assoc
NLM Journal ID: 101229936
Journal ID: JCHLA

The Journal of the Canadian Health Libraries Association

EISSN: 1708-6892
Publisher: Canadian Health Libraries Association / Association des bibliotèques de la santé du Canada

ARTICLE INFORMATION
==============================
Subjects: PP = Poster Presentation

PP8. Supporting primary care clinicians with evidence-based digital tools

Moore Taylor
Centre for Effective Practice

